# Insects perceive local sex ratio in the absence of tactile or visual sex-specific cues

**DOI:** 10.1007/s00265-012-1382-8

**Published:** 2012-07-20

**Authors:** Chang S. Han, Chang-Ku Kang, Hong-Sup Shin, Jeong-Hyun Lee, Mi-Rye Bae, Sang-Im Lee, Piotr G. Jablonski

**Affiliations:** 1 Evolution and Ecology Research Centre, School of Biological, Earth and Environmental Sciences, The University of New South Wales, Sydney, NSW Australia; 2 Laboratory of Behavioral Ecology and Evolution, School of Biological Sciences, Seoul National University, Seoul, South Korea; 3 Laboratory of Behavior and Ecology, Division of EcoScience, Ewha Womans University, Seoul, South Korea; 4 Institute of Advanced Machinery and Design, Seoul National University, Seoul, South Korea; 5 Center for Ecological Research, Polish Academy of Sciences, Dziekanow Lesny, 05 092 Lomianki Poland

**Keywords:** Sex ratio, Chemical cues, Ripple signals, Mating interactions, Sex recognition

## Abstract

Numerous studies have demonstrated adaptive behavioral responses of males and females to changes in operational sex ratio (the ratio of potentially receptive males to receptive females; OSR), and theory often assumes that animals have perfect instantaneous knowledge about the OSR. However, the role of sensory mechanisms in monitoring the local sex ratio by animals and whether animals can perceive local sex ratio in a manner consistent with model assumptions have not been well addressed. Here, we show that mating water striders *Gerris gracilicornis* respond to local sex ratio even when visual and physical contact with other individuals were experimentally prohibited. Our study shows that insects are able to estimate local population’s sex ratio and adjust their behavior based on nonvisual cues perceived at a distance or released to the habitat. Hence, the frequent theoretical assumption that individuals have knowledge about their local sex ratio regardless of their direct behavioral interactions may be an acceptable approximation of reality.

## Introduction

Animals adaptively respond to changes in operational sex ratio, the ratio of potentially receptive males to receptive females (OSR; Lawrence 1986; Jablonski and Vepsalainen 1995; Vepsalainen and Savolainen 1995; Weatherhead et al. 1995; Alonso-Pimentel and Papaj 1996), and theory often assumes that animals have perfect instantaneous knowledge about the OSR (Clutton-Brock and Parker 1992; Owens and Thompson 1994). However, the questions of whether animals can perceive local sex ratio in a manner consistent with model assumptions and how they do it have not been well addressed. It is possible that cues used to distinguish between sexes can also be used for monitoring the OSR. Animals can distinguish between sexes using visual (Rutowski 1977), acoustic (Miller 1979; Lind et al. 1996), chemical (Wyatt 2003), or vibrational signals (Wilcox 1979; Warren et al. 2009). For example, two-spotted ladybird beetle can discriminate sex by sex-specific behavioral cue (Hemptinne et al. 1998). Crickets (Hardy and Shaw 1983; Tregenza and Wedell 1997; Ryan and Sakaluk 2009) or beetles (Coleoptera; Peschke and Metzler 1987; Fukaya et al. 1996; Hemptinne et al. 1998; Ginzel et al. 2003; Zhang et al. 2003; Mutis et al. 2009) are able to recognize the sex with the chemical cue from the cuticular hydrocarbons. However, it is unknown whether these mechanisms are used by individuals to perceive and monitor the local OSR.

Water striders have been used as model organisms for evolutionary analyses of adaptive responses to the OSR. For example, researchers understand relatively well the evolutionary mechanisms responsible for the effect of OSR on female’s behavioral resistance to male copulation attempts or on the male postcopulatory mate-guarding behavior (Arnqvist 1992; Rowe 1992; Krupa and Sih 1993; Jablonski and Vepsalainen 1995; Vepsalainen and Savolainen 1995). The presence of such effects of OSR indicates that water striders are able to detect cues associated with OSR. Males should be able to do that even when they mate-guard on top of a female and their opportunities to monitor OSR through interactions with other individuals are limited (Jablonski and Vepsalainen 1995). Therefore, we hypothesized that, in addition to direct physical interactions, visual, chemical, or vibratory signals may serve as the cues for local OSR perception in water striders. For example, some water striders use sex-specific ripple signals to distinguish between the sexes (Wilcox 1979). Chemical cues are also used by Heteropteran insects to distinguish between sexes (Aldrich 1988) and the metathoracic scent gland is especially well developed in subfamily Gerrinae (Andersen 1982). This suggests that ripple signals and chemical cues may provide information about the local OSR to the water striders.

Before conducting experiments to determine which of the cues (from chemicals or vibratory signals), if any, are used by the water striders, we need to ascertain that these insects are able to detect OSR at a distance, without visual and tactile cues during direct interactions between individuals. The aim of this study was to determine whether the effect of OSR on mating behaviors can be observed when direct interactions between individuals and exchange of visual information are experimentally prohibited.

## Material and methods

### Study subjects

Males and females of *Gerris gracilicornis* were collected in Gwanak Mountain near Seoul National University, Seoul, South Korea, between 24 April and 14 May 2007. For at least 7 days prior to the test, we separated the water striders according to their sex (30 individuals per 30 × 40 cm container filled with water) in order to maintain a similar level of sperm storage in males before the experiment and in order to avoid variation among females in the amount of sperm already received through recent copulations. Frozen crickets (*Verlarifictorus asperses*) were given as the food, and pieces of floating Styrofoam were provided as the resting sites. In order to minimize the effects of body size on the mating behavior, we used individuals of similar body size, between the first and third quartiles of body length distribution (male, 11.9–12.6 mm; female, 14.1–14.8 mm).

### Experimental setup

We set four experimental treatments. Twenty containers (22 × 15 cm) were used for the male-biased sex ratio treatment (male/female = 4:1), and 18 containers were used for the female-biased sex ratio (male/female = 1:4). In ten containers for each treatment, we put two parallel partitions across the middle of each container just above the water surface (about 3 mm above the surface) to allow transmission of ripples on the surface of water across the partition. Also, the space between two partitions (2 cm) was created to prevent physical contact between the focal mating pair and the “background” three males or three females (depending on the treatment) located on the opposite side of the partition. Hence, we conducted ten replicates of *male-biased partition-present*, *male-biased partition-absent*, and *female-biased partition-present* treatments and eight replicates of *female-biased partition-absent* treatment. Each individual was used only once in the experiments.

### Behavioral variables and statistical methods

In this species, a mating interaction lasts many hours, with repeated copulation and guarding phases while the male remains on the female (Han et al. 2010). Therefore, any subsequent mating behaviors, except the very initial copulation, may be equally affected by the OSR as well as the preceding interactions within a pair. Considering this possibility, we chose to analyze the behavior of individuals only at the initial stage of a mating interaction. We chose two simple behavioral variables known to be affected by OSR in water striders (Rowe 1992; Krupa and Sih 1993; Jablonski and Vepsalainen 1995; Vepsalainen and Savolainen 1995): the female resistance to mating attempts and the copulation duration. Female *G. gracilicornis* “resists” to male’s copulation attempts by pushing away the male with her legs or by jumping. We recorded the presence or absence of female resistance during the mating initiation phase (i.e., from the time the focal pair started to interact until the copulation proper started as indicated by the extension of female genitalia; Han and Jablonski 2009). Exact logistic test (PROC LOGISTIC; SAS Institute 2002), which can handle contingency tables with zero cells (Stokes et al. 2000), was used to investigate the effect of two categorical independent variables, the OSR treatment (male-biased or female-biased) and the presence of partition (present or absent), on the presence of female resistance.

After copulation (intromission) started, we measured the duration (in seconds) of the first copulation bout (after which a male remains on a female in a guarding position). Since the copulation of *G. gracilicornis* was usually not terminated by females’ resistance but by the genitalia detachment by males mounted on the female (Han, personal observation), we regarded that the copulation duration is determined by the males. We missed the copulation termination of one male. Analysis of variance (ANOVA) was used to examine the effect of OSR and the presence of partition on the duration of the first copulation.

## Results

**Fig. 1:**
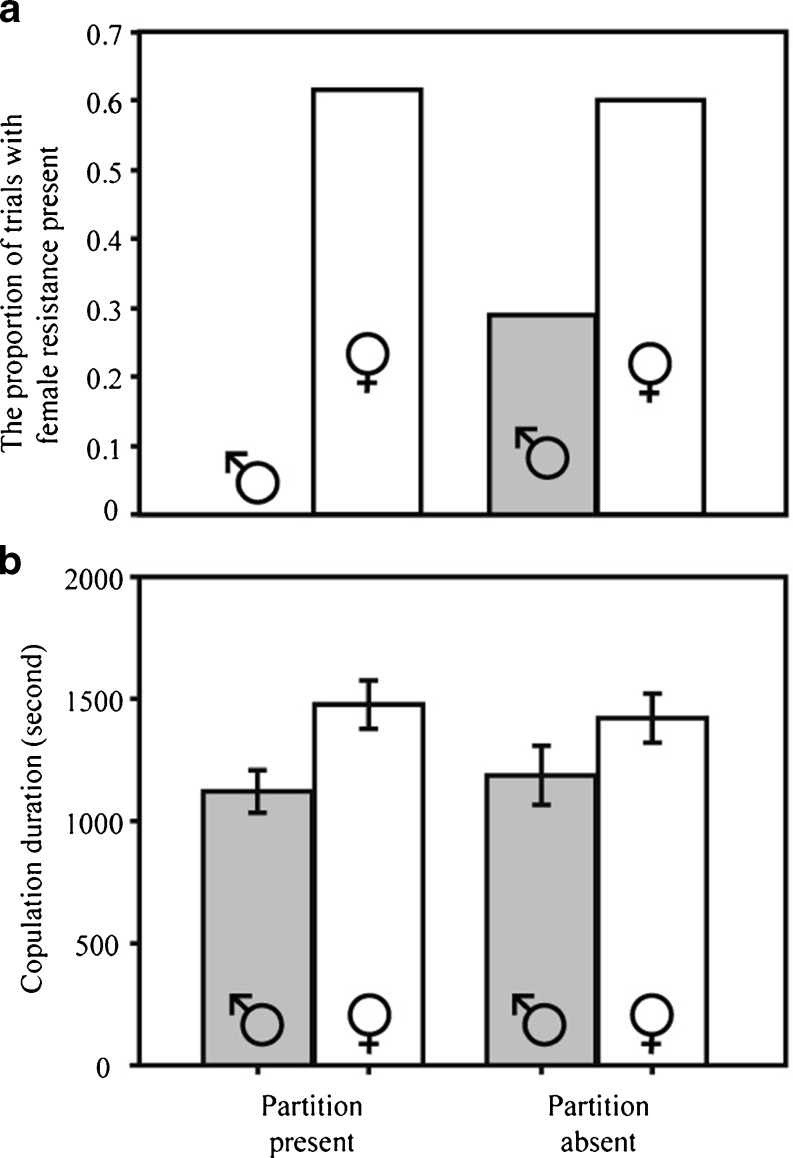
Effect of OSR (female-biased or male-biased; indicated as symbols) and the presence of an experimental partition that blocks direct physical interactions and visual cues on the mating behavior of females (**a**) and males (**b**) in *G. gracilicornis*. Means and standard errors are shown in **b**

More females resisted copulation in the female-biased than in the male-biased sex ratio (Table 1; test score = 8.90, *p* = 0.005) regardless of the presence or absence of the partition (interaction term not significant in Table 1). The results suggest that females recognized cues related to the OSR even in the partition-present treatment when no physical or visual contact was possible. Although the interaction was not significant, the graph (Fig. 1a) suggests that females might have reacted to the OSR in a more extreme manner when no physical interactions were allowed with other individuals in the population.

**Table 1:** Exact logistic test on the proportion of resistant females

Source	Test score	*p* value
OSR	8.90	0.005
Partition	1.94	0.26
OSR × partition	2.22	0.22

Copulation duration, determined by a male withdrawing his genitalia, was significantly affected by the OSR regardless of the presence of a partition (Table 2; Fig. 1b). Males copulated longer in female-biased than in the male-biased sex ratio treatment.

**Table 2:** Results of the ANOVA on the copulation duration of males

Source	*df*	MS	*F*	*p* value
OSR	1	817,671	8.03	<0.01
Partition	1	259	<0.01	0.96
OSR × partition	1	34,735	0.34	0.56
Error	34	101,824		

## Discussion

We showed that insects respond to local OSR in the absence of direct physical interactions or visual cues. The observed consistent behavioral responses to OSR regardless of the presence of the experimental partition clearly indicate that direct physical and visual cues are not needed to perceive the local OSR of a local population. While the proximate mechanism to recognize others’ sex at a distance has been reported in many insect taxa (Hardy and Shaw 1983; Peschke and Metzler 1987; Fukaya et al. 1996; Tregenza and Wedell 1997; Hemptinne et al. 1998; Ginzel et al. 2003; Zhang et al. 2003; Mutis et al. 2009; Ryan and Sakaluk 2009), the mechanism to monitor OSR was unclear because previous studies have not investigated whether individuals could monitor local OSR through such sex-specific cues.

What specific cues did the water striders use to determine the OSR? We considered that, in our experimental situation, males and females might have recognized the OSR using either the ripples on the water surface or chemical cues from other individuals. Water striders perceive ripples of the water surface through tarsal vibration receptors on their legs (Murphey 1971; Lawry 1973; Goodwyn et al. 2009). Females, even in the mating position, can receive cues from both the surface ripples and chemicals from other individuals in the population (through air or water as the medium). Males, however, have fewer opportunities than females to perceive ripple signals when they are in a mating position because mounted males’ forelegs and middle legs do not touch the water surface and their hindlegs are often out of touch with the water surface. Both sexes did not receive sex-specific ripple signals from other individuals in the experimental container because the sex-specific ripple signals, described in some water striders (Wilcox 1979), are not known in our study species *G. gracilicornis* (Han and Jablonski 2009). Furthermore, because *G. gracilicornis* males produce courtship signals at the very initial phase of a mating attempt (before intromission; Han and Jablonski 2009, 2010) and no additional mating pair (source of such signals) was present in the experiment, males and females could not have used the courtship ripples to estimate local OSR. The focal pair was the only mating pair in the experimental container and the only ripples on the water surface produced by the other experimental water striders (other than the focal pair) were due to their movements.

Because it seems unlikely that mating water striders might have perceived some information about OSR from simple ripples produced by moving water striders, we believe that they used chemical cues. The hypothetical role of chemicals in OSR monitoring is more strongly suggested by the fact that family Gerridae has the metathoracic scent gland, which is especially well developed in subfamily Gerrinae (Andersen 1982). These glands, located in the ventral part of the thorax, were suggested to have a role in sexual activity rather than in waterproofing of legs (Staddon 1979). Additionally, females of the marine water striders *Halobates hawaiiensis* were attracted to the extract from males consisting of male-specific palmitic and oleic acids, which disperse through the water surface rather than through the air (Tsoukatou et al. 2001). Therefore, we hypothesize that the scent glands in the genus *Gerris* may help in sex recognition and, thus, in perception of local OSR. Hence, our results not only suggest the need for future experiments to precisely determine the cues used by water striders in perceiving local OSR, but they also strongly indicate that chemical rather than vibrational cues are involved.

The pattern of water striders’ mating behavior in sex-biased conditions was consistent with our prediction and results from previous studies. Less resistant females in the male-biased sex ratio condition can be explained by “convenience polyandry” to avoid males’ harassment (Thornhill and Alcock 1983; Wilcox 1984; Rowe 1992). According to this mechanism, the cost associated with resistance and harassment from males is larger than the cost associated with multiple mating, and water strider females are less resistant to mating in male-biased sex ratio.

However, it is intriguing that water strider males copulated longer in the female-biased than in the male-biased sex ratio treatment. It has been known that males guard females longer in male-biased OSR (Clark 1988; Arnqvist 1992; Rowe 1992; Krupa and Sih 1993; Jablonski and Vepsalainen 1995; Vepsalainen and Savolainen 1995). Because of the prolonged mating in our study species, we could not measure guarding duration or any consecutive behavioral interactions precisely. But since *G. gracilicornis* males control copulation duration by withdrawing their genitalia (Han, personal observation), we could use the first copulation as the first behavioral response of a male to local sex ratio. Here, we propose a hypothesis that is consistent with the results. In the reproductive period, *G. gracilicornis* males are very aggressive and responsive to single females. Males frequently mount even on mating pairs, harass them, and sometimes disrupt sperm transfer of the mounting male and take over the female. We suspect that aggressive harassment of single males may disrupt the transfer of sperm of the mounting males by interrupting mounting male positioning on the female. Thus, because of the takeover or disruption of efficient sperm transfer, males in male-biased sex ratio may decrease the amount of sperm transfer and finish the copulation in a shorter duration.

We also considered an alternative explanation of why males copulated for longer in the female-biased OSR. Females were more resistant in this treatment. If stronger precopulatory resistance affects the efficiency of sperm transfer during the consecutive copulation, then in female-biased treatments, it might have taken longer for males to transfer sperm. However, we regard this hypothesis unlikely because a significant difference in female resistance between partition-present and partition-absent treatments in the male-biased condition (*G* = 4.7, *df* = 1, *p* = 0.03; Fig. 1) was not associated by a corresponding difference in male copulation duration (Tukey’s honestly significant difference (HSD) test, *p* = 0.97; Fig. 1).

Our results confirmed that adult insects detect population sex ratio without tactile or visual cues from other individuals. This brings the idea that juveniles may also be able to recognize demographic factors from nonvisual cues, such as sex-specific chemical compounds. The recognition of demographic factors such as sex ratio or density at a distance is very important for developing juvenile insects. Perceiving the social environment during development, juveniles can allocate the resources into traits suitable for the environment. For example, in a wing-dimorphic insect species, juvenile males in a male-biased condition may develop wings and disperse to a female-biased environment where males can encounter more females. However, because of the predation risk and limited mobility, juveniles have a difficulty to collect information on the local sex ratio by tactile or visual cues. If juveniles are able to infer the density or sex ratio of the population from chemical cues, they could use this information to modify their developmental pathways and to allocate their resources into traits that will maximize their fitness in specific conditions (Kasumovic and Brooks 2011). Hence, future studies may show that chemicals are reliable cues even for juvenile insects to gain information on demographic factors and to change their allocation strategy (Kasumovic and Andrade 2006; Kasumovic et al. 2009).

In summary, the frequent theoretical assumption that individuals have knowledge about their local OSR regardless of their direct behavioral interactions may be an acceptable approximation of reality. The results showed that water striders can perceive information about local OSR using cues that do not involve direct interactions, sex-specific vibratory signals, or visual information. Instead, they may use the cues that are released to the habitat (most likely sex-specific chemicals). Future experiments to precisely determine the hypothetical chemical cues used by those insects are needed.
